# Compare in-person and online outpatient visits based on changes in patients’ treatment

**DOI:** 10.1192/j.eurpsy.2022.1464

**Published:** 2022-09-01

**Authors:** K. Szczygieł, P. Podwalski

**Affiliations:** Pomeranian Medical University, Department Of Psychiatry, Szczecin, Poland

**Keywords:** e-mental health, telepsychiatry

## Abstract

**Introduction:**

The COVID epidemic has forced psychiatrists to introduce changes in outpatient clinics. A significant proportion of visits began without the patient’s face-to-face contact with the doctor. Are these visits stigmatized with a worse assessment of mental state? We know that much of the information flow takes place outside of verbal contact. In telephone contact, psychiatrists are limited to listening to the patient’s response and we know that non-verbal speech does not always go hand in hand with words.

**Objectives:**

The aim of the study is to compare face-to-face visits with a psychiatrist with outpatient visits by telephone in terms of changes in the treatment applied by psychiatrists.

**Methods:**

The frequency of introducing changes in the current pharmacological treatment of patients was compared. Face-to-face visits to the outpatient clinic and visits where psychiatrists contacted patients via telephone were analyzed. Treatment change was defined as a reduction or increase in drug dose, drug discontinuation or the initiation of a new drug by a psychiatrist.

**Results:**

We assumed that visits without non-verbal contact do not provide as much information as direct visits. Consequently, patients who are often negative about the need to take medications over the phone will present themselves better to psychiatrists, thus the change in treatment will be used less frequently in this group. The results will be presented at the conference.

**Conclusions:**

Currently, various forms of psychiatric care are evolving to adapt to new needs. We should also be aware of the consequences of these changes.

**Disclosure:**

No significant relationships.

